# Rhodamine-RCA in vivo labeling guided laser capture microdissection of cancer functional angiogenic vessels in a murine squamous cell carcinoma mouse model

**DOI:** 10.1186/1476-4598-5-5

**Published:** 2006-02-03

**Authors:** Finie Hunter, Jianwu Xie, Cameron Trimble, Monica Bur, King CP Li

**Affiliations:** 1 Molecular Imaging Laboratory, Clinical Center, National Institutes of Health, Bethesda, MD 20892, USA

## Abstract

**Background:**

Cancer growth, invasion and metastasis are highly related to tumor-associated neovasculature. The presence and progression of endothelial cells in cancer is chaotic, unorganized, and angiogenic vessels are less functional. Therefore, not all markers appearing on the chaotic endothelial cells are accessible if a drug is given through the vascular route. Identifying endothelial cell markers from functional cancer angiogenic vessels will indicate the accessibility and potential efficacy of vascular targeted therapies.

**Results:**

In order to quickly and effectively identify endothelial cell markers on the functional and accessible tumor vessels, we developed a novel technique by which tumor angiogenic vessels are labeled in vivo followed by Laser Capture Microdissection of microscopically isolated endothelial cells for genomic screening. Female C3H mice (N = 5) with established SCCVII tumors were treated with Rhodamine-RCA lectin by tail vein injection, and after fluorescence microscopy showed a successful vasculature staining, LCM was then performed on frozen section tissue using the PixCell II instrument with CapSure HS caps under the Rhodamine filter. By this approach, the fluorescent angiogenic endothelial cells were successfully picked up. As a result, the total RNA concentration increased from an average of 33.4 ng/ul +/- 24.3 (mean +/- S.D.) to 1913.4 ng/ul +/- 164. Relatively pure RNA was retrieved from both endothelial and epithelial cells as indicated by the 260/280 ratios (range 2.22–2.47). RT-PCR and gene electrophoresis successfully detected CD31 and Beta-Actin molecules with minimal Keratin 19 expression, which served as the negative control.

**Conclusion:**

Our present study demonstrates that in vivo Rhodamine RCA angiogenic vessel labeling provided a practical approach to effectively guide functional endothelial cell isolation by laser capture microdissection with fluorescent microscopy, resulting in high quality RNA and pure samples of endothelial cells pooled for detecting genomic expression.

## Background

The advent of laser capture microdissection (LCM) technology has remarkably advanced cancer research. Developed in a collaborative effort between the National Institutes of Health and Arcturus Engineering in 1996, LCM has served as an invaluable tool for separating tumor cells from normal tissue within heterogeneous neoplasms to better characterize their differences [1,2]. By microscopically extrapolating intact tissue with a simple pulse of a low-power infrared laser, the "captured" cells are adhered to a thermoplastic cap. These representative samples then provide a snapshot into the mechanisms occurring in the microtumor environment, permitting the analysis of biological molecules such as RNA and DNA, which remain structurally uncompromised during the microdissection process [3]. Thereafter, LCM derived cells can be examined using downstream assays such as polymerase chain reaction (PCR) and quantitative, real-time PCR, providing much insight into the regulatory pathways observed within proliferating cancer cells. Several studies have utilized LCM to conduct molecular analysis of pure isolated cells from breast, ovarian, prostate and squamous cell carcinomas [4-8]. As a result, the application of LCM in research has created genomic and proteomic expression profiles and many cell-specific markers have been identified [9].

An innovative approach to cancer therapy has focused on angiogenic targeting of molecular markers on the luminal surface of tumor vasculature to promote tumor regression [10-13]. Proliferating endothelial cells are the prime targets of vascular targeting agents (VTAs) utilized for controlling angiogenesis, the process by which new blood vessels are formed [14]. For this purpose, efforts to classify and characterize tumor vasculature, specifically neovasculature, has prompted close examination of mosaic blood vessels, circulating endothelial cells and endothelial cell precursors [15]. These endothelial modalities integrate into new and existing blood vessels. In comparison to cancer cells, which develop resistance to anticancer agents, endothelial cells are of special interest and serve as better targets because they are less likely to mutate[16].

Angiogenesis researchers have extensively investigated the effects of naturally occurring antigens such as Alpha-V Beta-3 and vascular permeability factor (VEGF) on cell regulation. These inducers of angiogenesis which mediate numerous functions of endothelial cells including proliferation, migration, invasion, survival, and permeability have proven to be useful therapeutic targets for diseases characterized by neovascularization. Monoclonal antibody targeting as well as angiogenesis inhibitors have been utilized in clinical studies to cut off blood supply to targeted cells in tumors [17,18]. With this in mind, the application of laser capture microdissection is an ideal method for studying the effects of angiogenic inhibitors, identifying cell surface antigens for antibody targeting and discovering new biomarkers on tumor endothelium at molecular and genetic levels.

Typically, researchers have utilized immunohistochemical (IHC) and fluorescent staining against specific markers for endothelial cells such as von Willebrand Factor (VWF), CD34, CD31 and *Ricinus communis *agglutinin I (RCA) lectin to identify tumor vasculature and guide the laser capture microdissection process [19-22]. To date, several studies have focused on optimizing staining protocols to reduce the processing time of samples for LCM because evidence supports the notion that there is a direct correlation between RNA degradation and factors such as length of tissue processing time and exposure to RNases during sample preparation [23-25]. Murakami et al. reported that exposure to aqueous media for an extended period of time, a requirement of traditional immunohistochemical staining, could "destroy 99% of the mRNA" from a sample [26]. While rapid immunohistochemical and fluorescent staining can effectively label endothelial cells in vitro, these pathological tools are limited by their inability to identify and distinguish between functional and accessible tumor vasculature.

Therefore, we developed a novel methodology for intravenously labeling cancerous endothelial cells with fluorescent Rhodamine RCA lectin to guide LCM. By this approach, in vivo labeling circumvents the aforementioned technical issues while effectively identifying functional tumor vessels that are accessible if a drug was given via the vascular route. This method of endothelium labeling has not been previously combined with laser capture microdissection and provides a roadmap for molecular targeting in endothelial cells. Moreover, in vivo labeling of angiogenic vessels provides a practical technique for discovering surrogate markers of angiogenesis and a clinically relevant strategy for vascular targeted therapies. The objective of this study was to demonstrate the efficacy and compatibility of endothelial cell in vivo labeling with RCA lectin to further guide LCM for mRNA detection and quantitation. Here, we verify the reliability of RCA lectin binding to identify functional and angiogenic tumor vessels.

## Materials and methods

### 1) Cell culture

SCCVII, a murine squamous cell carcinoma cell line was kindly provided by Dr. Anastasia Sowers (Radiation Oncology Branch, National Cancer Institute, Bethesda, MD, USA) and cultured at 37°C with 5% CO_2 _in RPMI 1640 medium (Biofluids, Gaithersburg, MD, USA) containing 10% fetal calf serum, 2 mM L-glutamine, and penicillin-streptomycin (BioSource International; Rockville, MD, USA), 50 IU ml^-1 ^and 50 μg ml^-1 ^respectively.

### 2) Animal model and RCA *in vivo *labeling of tumor angiogenic vessels

Female C_3_H mice (6–9 weeks) were purchased from Charles River Laboratories (Gaithersburg, MD, USA) and subjected to S.C. inoculation. All animal work was performed according to an approved animal protocol and in compliance of NIH Clinical Center Animal Care and Use Committee guidelines. A suspension of SCCVII cells (1 × 10^6 ^cells in 100 μl of PBS) was injected in the left flank of five mice with a 27 G needle. The inoculated mice were monitored daily for growth of the tumors; the tumor volume being measured every second day using calipers. On average, a tumor required 7–10 days to grow to 0.4 cm^3 ^in volume when the tissue was ready for harvesting. Technically, *in vivo *labeling of the functional tumor vasculature was performed via tail-vein injection of Rhodamine *Ricinus communis agglutinin I *(RCA) (Vector Laboratories, CA, USA) and allowed to circulate for 5 min before the mice were euthanized. Immediately after the mice were sacrificed, the tumors were harvested, quickly frozen on dry ice and stored in -80°C until processing.

### 3) RNAase free sample preparation

To prevent any contamination of RNases, the Leica Cryostat Instrument was cleaned with 100% ethanol and a new disposable blade was replaced prior to sectioning the tumor samples. Each frozen tumor sample was removed from the cryovial using clean forceps, immediately embedded in OCT medium (Thermo Shandon, PA, USA) and allowed to equilibrate to the cryostat temperature of -21°C. Fifty tissue sections for each of the five samples were sectioned at 7 μm, placed on Histogene™ LCM Slides (Arturus, CA, USA) and directly fixed in fresh cold acetone (+4°C) for 2 minutes. Two slides per LCM session were completely dehydrated in sterile jars (Evergreen Scientific, CA, USA) containing RNAase free reagents provided in the Histogene Dehydrations Kit (Arturus, CA). Each slide was placed in solutions of 75% ethanol-30 sec, 95% ethanol- 30 sec, 100% ethanol-30 sec, and Xylene- 5 minutes respectively and allowed to dry for five minutes on a horizontal staining rack.

### 4) Laser capture microdissection

RNase free conditions were maintained by wiping the PixCell II LCM system (Arturus, CA, USA) stage and work areas surrounding the instrument with RNase Away. Immediately following dehydration, the tissue was examined under fluorescent microscopy to detect the successful tumor angiogenic vessel staining under the Rhodamine filter. Then, the parameters were established for selection and excision of endothelial cells with at least a 6 μm diameter. In order to achieve a laser spot size smaller than 7.5 μm, which is the minimum size reported by Arcturus, we determined that a power setting of 40 mW and duration of 450 μs was sufficient. Only the vessels propagating fluorescence were chosen and pulsed by the manually maneuvered laser spot. By this approach, fluorescent labeled vessels were detached from the slide and lifted onto a thermoplastic polymer film on the high sensitivity (HS) Cap. Within a one hour time frame per slide, we were able to collect approximately 150–200 endothelial cells per sample. Epithelial cells were also microdissected under the following laser conditions: 15 μm spot size, power 50 mW and at 2.0 W duration. Four HS caps, generating a total of 600–800 endothelial cells for each of the five tissue samples were collected and pooled to generate an adequate concentration of RNA for downstream analysis.

### 5) RNA analysis and quality assessment

Processing of the total RNA began immediately following LCM. Extraction, isolation and two rounds of amplification were performed using the PicoPure RNA Isolation Kit (Arcturus, CA, USA) and the RiboAmp HS RNA Amplification Kit (Arcturus, CA, USA) respectively, according to the manufacturer's recommendations. Exactly 10 μl of extraction buffer was added to the ExtractSure device, in which the samples remained on the HS cap positioned upright in the heating block. Each aperture was covered with a 0.5 ml- Eppendorf tube (Arcturus, CA, USA) and incubated at 42°C for 30 min, followed by centrifugation at 800 × g for two minutes. At that point, all endothelial cell pooled samples were combined for isolation, and a net yield of 11 μl was quantified using UV Spectrophotometry with Ribogreen Fluorescence. Next, mRNA was generated after two rounds of amplification.

### 6) Reverse transcription-polymerase chain reaction

First strand cDNA was synthesized with the Promega Reverse Transcription System according to the manufacturer's recommendations. The reverse transcription reaction was primed with random primers, starting from 5'end throughout the length of the RNA. The first of the two 10 μl mixtures were prepared containing 2 μl of RNA sample, denatured at 70°C to prevent secondary binding, and 8 μl DEPC treated water. The second mixture consisted of 4 μl MgCL_2_, 2 μl RT 10 × Buffer, 2 μl dNTP, 0.5 RNase inhibitor, 1 μl AMV RT Transcriptase and 0.5 μl random primers. Both mixtures were combined and reverse transcription was conducted at the following settings: 20°C ~10 min, 42°C~45 min, 95°C ~5 min, and 4°C ~∞ in the AB9700 Thermocycler. (Applied Biosystems, CA, USA). Thereafter, cDNA was stored at -20°C overnight.

### 7) Polymerase chain reaction

Three microliters of cDNA was combined with a 47 μl PCR mix consisting of 5 μl 10 × buffer, 1 μl DNTP, 0.3 μl TAQ polymerase, 38.7 μl Ultra Pure Water and 1 μl from each set of primers. Messenger RNA sequences for CD31, KER19, Beta-Actin were custom designed for our experimentation to determine the purity of the LCM samples. Beta Actin primers as P1: 5'CGTGGGCCGCCCTAGGCACCA-3', P2: 5'-TTGGCCTTAGGGTTCAGGGGGG-3'; CD31 primers: P1 5'-GTGCTCTATGCAAGCCTCCA-3', P2: 5'-TTCGAGGTGGTGC TGATGTC-3', and Mouse Cytokeratin 19 primers P1: 5'-TG GGCAGATTGTTGTTATGGA-3', P2: 5'-TCAGGGCAGTAATTTCCTCC-3' as seen in table 1. The PCR Core Kit was utilized to synthesize dsDNA sequenced at 60 cycles at 94°C ~30 sec, 55°C ~30 sec and 72°C for 45 sec for denaturation, primer annealing and extension of annealed primers respectively.

**Table 1:** mRNA custom designed primer sequences for polymerase chain reaction analysis of angiogenic endothelial cells isolated from SCCVII mouse model neoplasms.

Gene		Sense sequence		Anti-sense sequence
GADPH	P1	5'-ACCACAGTCCATGCCATCAC-3'	P2	5'-TCCACCACCCTGTTGCTGTA-3'
Beta Actin	P1	5'-CGTGGGCCGCCCTAGGCACCA-3'	P2	5'-TTGGCCTTAGGGTTCAGGGGGG-3'
CD31	P1	5'-GTGCTCTATGCAAGCCTCCA-3'	P2	5'-TTCGAGGTGGTGCTGATGTC-3'
	P3	5'-ATGGAATTCCCCATTGAGGC-3'	P4	5'-GATTTCAAACTTGGGAGTGG-3'
	P5	5'-TTCTGAACTCCAACAGCGAG-3'	P6	5'-AGGCTCAAGGGAGGACACTT-3'
	P7	5'-TGCCGAAGGCCCAAAGAAGA-3'	P8	5'-GCTCAGACCTTAGGAAACCG-3'
Keratin 19	P1	5'-TGGGCAGATTGTTGTTATGGA-3'	P2	5'-TCAGGGCAGTAATTTCCTCC-3'
	P3	5'-CCTGGCTGCAGATGACTTCA-3'	P4	5'-AGACGGAGGCCCGTTATGGA-3'
	P5	5'-TAAAACTTCCACCGCGGACT-3'	P6	5'-TTCCTATAGCTATCGCCAGA-3'
Alpha V	P1	5'-CTCTGAATCTGCCGTCAGT-3'	P2	5'-GCCAAGTGCTTGCAGATCAC-3'
	P3	5'-GTGATGTTAGTGGTGACTAG-3'	P4	5'-AAGGTTGACCTCGCCGAAAG-3'
Beta 3	P1	5'-CTGTTACAATATGAAGAATG-3'	P2	5'-TTTTCATCACATACTGTAGC-3'
	P3	5'-ACTGTGCCTGCCAGGCCTTT-3'	P4	5'-TCCTTGGGGCTGCACTCTTC-3'

## Results and discussion

### Identifying functional tumor vasculature for LCM with RCA-I lectin

Intravascular labeling and immunohistochemical staining of endothelial cells with lectin are relatively established methods. Ricinus communis agglutinin I (RCA-I), Ulex europaeus agglutinin-I (UEA-I) and Wheat germ agglutinin (WGA) have been recognized as practical markers for endothelial cells in intact tissue and cell culture. Gerhart et al. reported strong staining of RCA-I in vessels of canine cerebral cortex and capillaries bound to luminal membrane of endothelial cells [27]. Their results indicated that lectin receptors were distributed continuously along the endothelial membrane. Likewise, Chang et al. utilized Rhodamine- RCA lectin as a perfusion marker to identify functional blood vessels in Enhanced Green Fluorescent Protein (EGFP) expressing tumors [28]. Equally important, their feasible and reliable technique of intravenously labeling endothelium provided no evidence of nonperfused vessels. Systemic RCA-I labeling of functional tumor vasculature utilizes targeted lectin-carbohydrate based interactions to identify receptors uniquely expressed on endothelial cell surfaces. For these reasons, we modeled this method of systemic injection for labeling functional and angiogenic tumor vessels to guide laser capture microdissection. As a result, we successfully observed all accessible and functional endothelial cells labeled with RCA-I under fluorescent microscopy.

### Significance of in vivo labeling of tumor vasculature

**Figure 1:**
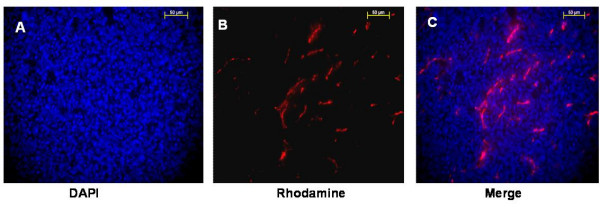
**RCA lectin *in vivo *labeling is an effective way to mark functional angiogenic vessels**. SCCVII tumors were systemically labeled with Rhodamine-RCA Lectin and 7 μm tissue sections were counterstained with DAPI and observed under 40× objective upright microscope (Zeiss, USA) through Rhodamine and DAPI filters. (scale bar = 50 um) **A: **Endothelial cell nuclei staining pattern observed with bright blue DAPI counterstain in SCCVII neoplasm. **B: **Functional angiogenic vessels labeled by tail vein injection of Rd-RCA lectin. RCA-I is able to successfully stain endothelial cells due to the specific affinity to beta D-galactose which is found on the luminal surface of blood vessels. **C: **Merged signals of vessels and the cell nuclear staining by DAPI. The abundance of nuclei present in the tissue is not representative of the functional and accessible tumor vasculature in the merged image.

A new technique in vascular targeting, such as physically isolating host endothelium, is an ideal strategy for discovering molecular markers with tumor specificity. Our present study was founded on the hypothesis that the phenotypes of in vivo labeled endothelial cells and IHC stained endothelial cells are distinct. Although RCA lectin immunohistochemical staining could successfully label the tumor vasculature, not all vessels expressing endothelial markers are accessible to drugs administered systemically. Consequently, immunohistochemical staining would yield remote gene profiling, unrepresentative to that of the proliferating cells. The significance of in vivo labeling was demonstrated by comparing the quantity of functional tumor vasculature identified by systemic injection of RCA versus IHC staining. As seen in Figure 1A, a plethora of DAPI stained nuclei was observed in the tumor, however, an overlapping image of RCA-I labeled vessels within the same tissue (Figure 1C) revealed that the vast number of nuclei detected by IHC was not representative of the few functional vessels present in the tumor section.

### Laser capture microdissection of functional endothelial cells

**Figure 2:**
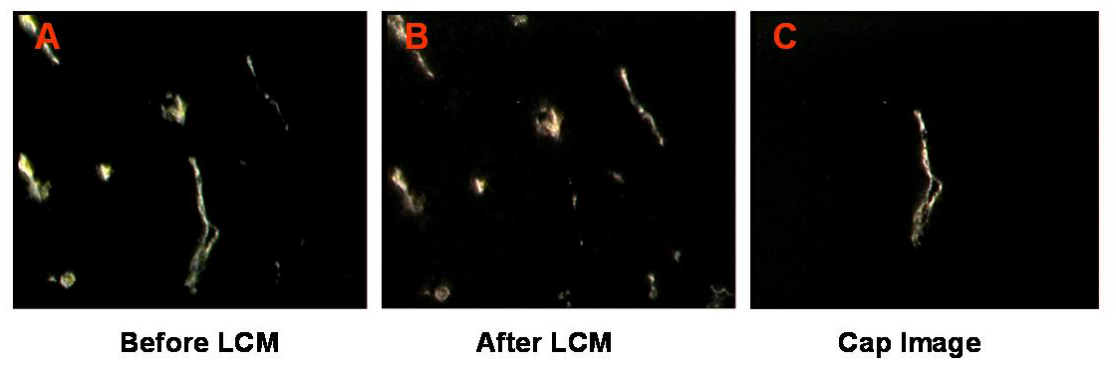
**Successful Rd-RCA I lectin guided capture of endothelial cells under inverted 40× magnification. A: **Fluorescent labeled angiogenic and functional vessels are distinguished from the surrounding epithelium prior to performing LCM. **B: **A low-powered infrared laser was pulsed over the targeted cells, which expanded the film on the cap and extracted the entire vessel. **C: **An intact vessel containing a homogeneous cell population generated for molecular analysis. The presence of RCA-Rhodamine labeled endothelial cells on the "cap" image and the absence of the targeted cells in the "after" microdissection image confirmed successful capture. A power setting of 40 mW and duration of 450 μs were utilized to obtain a 5.0–6.0 μm diameter laser spot size, which permitted precise selection of endothelium using the Pix Cell II Instrument.

While LCM provides a simplistic yet comprehensive approach for isolating select cells from a variety of tissue types, procurement of endothelial cells can be quite challenging. There are several technical issues that must be considered when optimizing LCM protocols to microdissect mouse endothelium. First, in order for LCM to work effectively the targeted endothelial cells must be distinguished from surrounding epithelium to yield a homogenous cell population. This matter was addressed by intravenously labeling the tumor vasculature with RCA-I lectin (Figure 2A). Second, modifications to the laser spot size were necessary to avoid extracting adjacent pericytes, which are also present in capillary walls. Since, tumor vessels vary in diameter from 1–8 μm and the smallest standard spot size for the LCM instrument is 7 μm, we adjusted the laser spot according to a reported method for controlling the spot size [29,30]. By decreasing the laser power, we precisely removed thin tumor vasculature from the host environment (Figure 2B, 2C). The third concern involved generating enough tissue samples on each cap to retrieve sufficient mRNA for downstream assays. Due to the limitations of the laser spot size, only 2–3 cells were captured per laser pulse.

Consequently, it was necessary to pool four caps to produce an adequate concentration of starting total RNA for each of the five endothelial cell samples as described by Zhang et al [31]. Overall, the optimized protocol permitted successful extraction of SCCVII mouse model endothelial cells.

### Determining the quality and concentration of RNA

**Figure 3:**
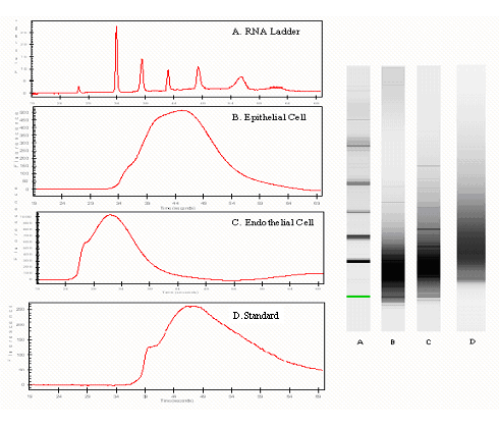
**Assessment of RNA integrity in frozen sections of mouse SCCVII tumor tissue. **The Agilent BioAnalyzer system determined that the RNA from two-round amplification of endothelial and epithelial cell samples had comparable concentration of 1278.5 ng/μl. The spectra clearly indicated the presence of a marker peak and a broad hump in the electropherogram confirming good RNA quality. Further mRNA analysis demonstrated a high quality starting sample of total RNA by the 260/280 ratio for the five endothelial and epithelial cell samples. Moreover, the RNA quality and concentration was comparable to that of the control sample.

The BioAnalyzer provided a general quality assessment of the LCM derived samples; however we relied on the NanoDrop to provide a more precise measurement of the RNA extract's optical density. The electropherogram in Figure 3 confirmed that the amplified RNA was of good quality characterized by a broad hump. Contrarily, a spectrum of contaminated samples would display sharp peaks with high background and short integration time. The fluorescence reading revealed that the concentration of amplified epithelial cells exceeded that of the endothelial cell sample, which was understandable considering the large variation in the laser spot size when performing LCM. Since epithelial cells in cancer tissue are relatively abundant and easier to extract, we used a larger laser spot size to collect these samples. For experimentation purposes, the epithelial cells only served as a control to determine the purity of the endothelial cells. Therefore, epithelial cell concentration at this level of analysis was not a major factor. A Ribogreen Low Range Calibration was performed to detect the amount of nucleic acid present in the isolated vessel tissues.

The initial RNA concentration of microdissected samples was compared to the final sample concentration after performing two-round amplification. As a result cap pooling and amplification, the total RNA concentration increased from 33.4 ng/μl ± 24.3 (mean ± S.D.) to 1913.4 ng/μl ± 164 in the endothelial cell samples. Relatively pure RNA was retrieved from endothelial and epithelial cells as indicated by the 260/280 ratios (range 2.22–2.47). Exactly, 1 μl sample from all five samples of endothelial cells yielded a mean ratio of 2.25. Similarly, the mean ratio for the five epithelial cell samples was 2.21. Ratios lower than 1.8–2.0 are indicative of contamination. These results were comparable to that of the control samples (N = 5), which had a mean total RNA concentration of 1987.06 ng/μl ± 27.8 and an average 260/280 ratio of 2.58.

**Figure 4:**
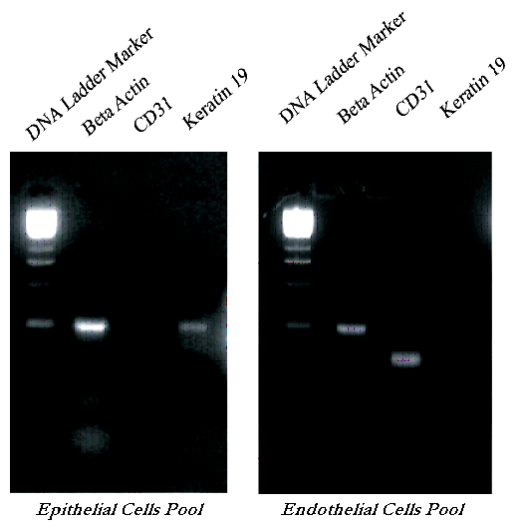
**The purity of endothelial cell RNA pool is measured by RT-PCR amplification of endothelial cell marker CD31, squamous cell carcinoma epithelial specific gene Keratin 19 as well as the Beta-Actin housekeeping gene from LCM samples**. Approximately 200 endothelial cells as well as control epithelial cells were microdissected and captured from each sample RNA was extracted and then amplified. In order to verify the quality and purity of the LCM endothelial RNA samples, custom designed primers for house keeping gene Beta-Actin, CD31D, and two pairs of Keratin 19 (KER19A, KER19 B) were applied to the pool by RT-PCR. The results indicated that the endothelial pool does not contain Keratin 19 mRNA, which is expressed in the epithelial pool.

We performed two-step RT-PCR, in which the RT reaction and PCR were sequentially conducted, utilizing two different sets of primers during the RT and PCR steps (Table 1). Random primers were used for general amplification throughout the mRNA template, followed by PCR with several pairs of gene-specific primers. Expression of CD31 in the LCM-derived endothelial cells was detected by RT-PCR in addition to using Beta-Actin as an internal control. Expression of Beta-Actin was conserved after two rounds of amplification. The absence of Keratin in the endothelial sample validated the accuracy of our technique, demonstrating that we extracted a pure endothelial cell sample. PCR products were visualized in a 2% agarose gel stained with ethidium bromide (Figure 4). In contrast, the epithelial cells showed positive Keratin expression Keratin, although it was downregulated. The low expression of Keratin in our epithelial sample may be attributed to the uniqueness of the cancer cell line used in the study. Our results were consistent with findings from the literature which reveal that Cytokeratin 19 is highly expressed in normal epithelium; however squamous cell carcinomas exhibit patchy negative staining [32]. Ultimately, the endothelial cells displayed upregulation of CD31 expression with minimal contamination.

### Significance of antibodies

While intravenous labeling of endothelium established that the targeted tumor vasculature was functional, still the angiogenic nature of the captured vessels had to be determined. For this purpose, we utilized gel electrophoresis to detect the presence of Alpha-V Beta-3 integrin. Alpha-V Beta-3 is a protein that is upregulated in various pathological conditions including tumors associated with angiogenic endothelial cells. In order to measure the sensitivity of our method, we selected CD31 as an endothelial marker and Keratin 19 as the epithelial cell marker. Positive immunohistochemical staining of CD31 in figure 4 confirmed that it was an excellent cell marker for endothelial cell recognition. Also, Keratin 19, which is a squamous cell carcinoma epithelium marker served as the negative control and indicated epithelial contamination in LCM-derived probes [33]. Success of the compatibility of the in vivo labeling with laser capture microdissection was ultimately determined by the quality of mRNA retrieved from the LCM derived samples, expression of CD31 and house keeping genes Beta-Actin and GADPH. The absence of Keratin 19 in the endothelial cells demonstrated little or no contamination in the endothelial cell sample.

### Study limitations

The application of our in vivo labeling technique may be challenged by the fact that shortly after systemic administration of Rd-RCA lectin the mice were euthanized. Still, neither the perfusion of vessels or specificity of lectin binding to the tumor endothelium was affected by this procedure. Also, the toxicity of lectin is a major concern when considering the practicality of our in vivo labeling method for clinical application. We show unambiguous evidence that the moderate toxicity of RCA-I does not denature or alter the genetic material within the labeled and microdissected vasculature. Specifically, systemic RCA lectin labeling of tumor vasculature detected antibodies that are notably found in endothelial cells. Moreover, we demonstrated that endothelial cell phenotypes in SCCVII mouse model tumors can be discriminated by lectin binding.

### Practical applications

Preclinical studies have shown that the angiogenic switch occurs early in the multi-step stage of cancer development, which is required for tumor growth and metastasis [34]. The intercellular junctions of endothelial cells control the intake and release of local agents, which is an integral factor in therapeutic gene delivery. This methodology makes possible to study the factors that regulate angiogenesis progression and tumor regression.

In vivo labeling of endothelial cells using RCA-lectin can play a pivotal role in the discovery of new organ specific molecular markers and vascular targeted therapies. Ribatti et al. has suggested that one problem in defining organ-specific endothelial cell markers is due to the impurity of endothelial cell used for in vitro analysis and to the lack of organ specific markers of endothelial cells in culture [35]. Indeed, our study provides a solution in that laser capture microdissection guided by in vivo labeling of vasculature can produce homogenous cell populations from endothelial cells and could identify organ specific endothelial cell markers. Also, labeling of functional endothelium with lectin could provide information about cell-cell interactions within the host environment that contribute to the induction and maintenance of angiogenic factors. RCA binding may indicate that extracellular matrix components regulate endothelial cell surface D-galactosyl residues, lymphocytes and metastatic tumor cells. In vivo labeling of endothelial cells combined with LCM can provide information about the metastatic potential of angiogenic vessels based on the specificity of lectin binding. The parameters of this in vivo study, including the brief circulation period of RCA-lectin would be ideal for identifying functional and angiogenic vessels during tumor biopsies to better characterize the tissue.

## Conclusion

Our present study demonstrates that *in vivo *Rhodamine RCA angiogenic vessel labeling provided a practical approach to effectively guide functional endothelial cell isolation by laser capture microdissection with fluorescent microscopy, resulting in high quality RNA and pure samples of endothelial cells pooled for detecting genomic expression.

## Abbreviations

**IHC**, immunhistochemistry; **RCA**, *Ricinus communis agglutinin I*; **LCM**, laser capture microdissection; **RT-PCR**, reverse transcription-polymerase chain reaction.

**Figure 5:**
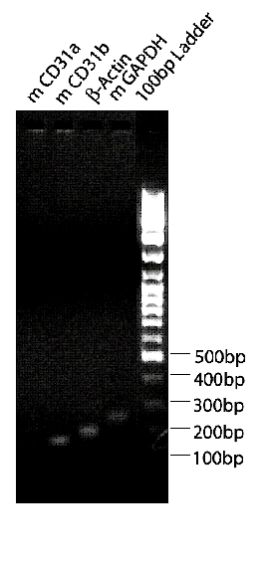
**RT-PCR amplification of housekeeping genes and CD31 endothelial cell markers from LCM derived cells. **About 200 endothelial cells were microdissected and captured, the RNA was extracted and amplified. After first strand cDNA synthesis, primers designed for murine CD31 RT-PCR **mCD31a: **5'TTCTGAACTCCAACAGCGAG-3', 5'-AGGCTCAAGGGAGGACACTT-3'; **mCD31b**: 5'-TGCCGAAGGCCCAAAGAAGA-3', 5'GCTCAGACCTTAGGAAACCG-3, GAPDH and β-Actin were detected by PCR.

**Figure 6:**
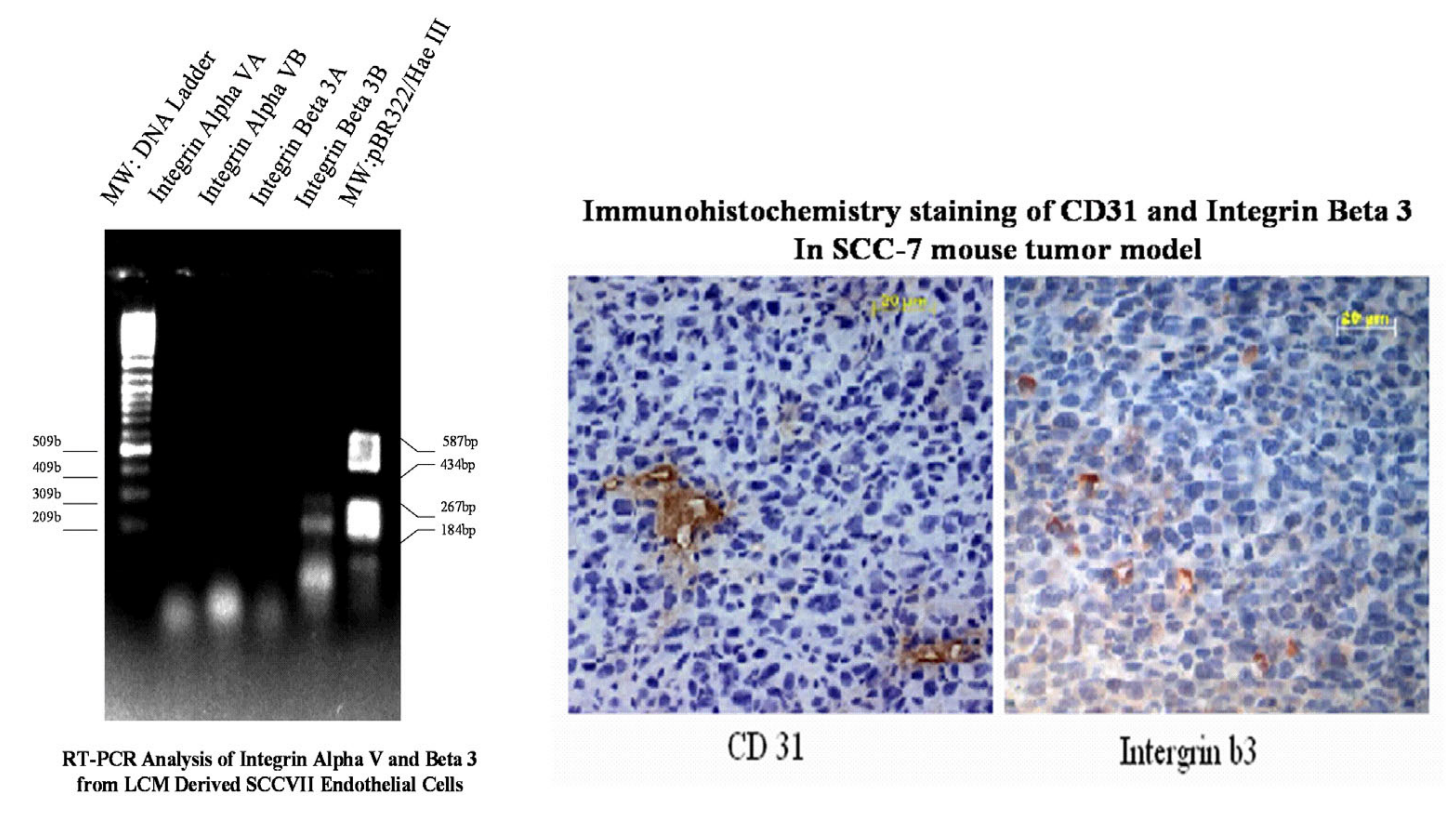
**Integrin Beta 3 and CD31 are highly expressed in the SCCVII tumor angiogenic vessels **detected by RT-PCR from laser capture microdissected endothelial cells and confirmed by immunohistochemical staining of Integrin Beta-3. Integrin immunohistochemistry on paraffin embedded 7 μm sections were stained with mouse monoclonal antibodies from BD-Pharmingen, USA at a dilution of 1:200. Positive CD31 and Beta 3 showed dark brown and intense orange-brown staining, respectively. Each tumor section was counterstained with hemotoxylin.
